# Real-world efficacy and safety of durvalumab–tremelimumab as second-line systemic therapy after atezolizumab–bevacizumab in unresectable hepatocellular carcinoma

**DOI:** 10.1097/MD.0000000000039289

**Published:** 2024-08-23

**Authors:** Ryoichi Miura, Atsushi Ono, Shigeki Yano, Kei Amioka, Kensuke Naruto, Kenji Yamaoka, Yasutoshi Fujii, Shinsuke Uchikawa, Hatsue Fujino, Takashi Nakahara, Eisuke Murakami, Tomokazu Kawaoka, Daiki Miki, Masataka Tsuge, C. Nelson Hayes, Shiro Oka

**Affiliations:** a Department of Gastroenterology, Hiroshima University Hospital, Hiroshima, Japan; b Department of Clinical Oncology, Graduate School of Biomedical and Health Sciences, Hiroshima University, Hiroshima, Japan.

**Keywords:** hepatocellular carcinoma, immune checkpoint inhibitor, immune-related adverse events, rechallenge, treatment efficacy

## Abstract

The efficacy and safety of immune-checkpoint inhibitors (ICI) for the treatment of unresectable hepatocellular carcinoma are known. We explored ICI rechallenges with direct switching from 1 ICI regimen to another. This retrospective study included 16 patients who received atezolizumab–bevacizumab (Atezo+Bev) and durvalumab–tremelimumab (Dur+Tre) as the first-line and second-line combination therapy, respectively, at Hiroshima University Hospital. The radiological response and adverse event were evaluated in all patients. Of the 16 patients, 12 were male, and the median age at Atezo+Bev induction was 71 years. The reasons for medication changes were disease progression in 11 patients and adverse events in 5 patients. With Atezo+Bev and Dur+Tre initiation, the Barcelona-Clinic Liver-Cancer stage (A/B/C) progressed in 9/6/3 and 3/4/9 patients and the Child–Pugh classification (A/B/C) progressed in 12/4/0 and 9/6/3 patients, respectively. The disease control rate and overall response rate of Atezo+Bev were 87.5% and 58.3%, respectively, and of Dur+Tre were 62.5% and 0%, respectively. The most common immune-related adverse event in both the Atezo+Bev and Dur+Tre groups was colitis; 3 of the 5 patients with colitis on Atezo+Bev treatment had colitis with Dur+Tre, and 2 had exacerbations. Regarding liver function, ALBI score significantly decreased during Atezo+Bev, but not Dur+Tre, treatment. In patients with colitis following Atezo+Bev, subsequent Dur+Tre treatment may induce colitis recurrence or exacerbation. For immune-related adverse events other than colitis, Dur+Tre could provide relatively safe disease control while maintaining liver function.

**Key Points**

This study evaluated the efficacy and safety of directly switching from atezolizumab–bevacizumab to durvalumab–tremelimumab for unresectable hepatocellular carcinoma in 16 patients based on their clinical course, outcomes, and side effects. Directly switching from atezolizumab–bevacizumab to durvalumab–tremelimumab is relatively safe and can achieve disease control, despite a risk of colitis exacerbation after durvalumab–tremelimumab initiation in patients with colitis during atezolizumab–bevacizumab treatment.

## 1. Introduction

Worldwide, primary liver cancer was the 6th most common cancer and the 3rd leading cause of cancer-related death in 2020. Hepatocellular carcinoma (HCC) is the most common histological type of primary liver cancer and accounts for 75% to 85% of all cases.^[1]^ Systemic therapy has an important role in treating unresectable HCC (uHCC), and multi-targeted tyrosine kinase inhibitors (TKI), such as sorafenib, have long been used in first-line treatment. In 2020, the IMbrave150 study, an open-label phase III trial, showed that the atezolizumab–bevacizumab (Atezo+Bev), an immune-checkpoint inhibitor (ICI)–immune-combination therapy, significantly improved overall survival (OS) and progression-free survival compared to sorafenib (median OS; Atezo+Bev vs sorafenib: 19.2 months vs 13.4 months), and a higher response was achieved with Atezo+Bev than sorafenib (overall response rate [ORR]; Atevo+Bev vs sorafenib: 27.3% vs 11.9%; *P* < .001),^[2]^ ICI-based regimens are now recognized as first-line agents. Several ICI-based regimens have been developed thus far. In 2022, the STRIDE study, an open-label, phase III trial that compared durvalumab–tremelimumab combination therapy (Dur+Tre) with sorafenib, showed improved survival (median OS; Dur+Tre vs sorafenib: 16.4 months vs 13.7 months; *P* < .0035) and thus confirmed the superiority of Dur+Tre compared to sorafenib.^[3]^ In Japan, based on the results of the IMbrave150 and STRIDE trials, 2 ICI regimens—Atezo+Bev and Dur+Tre—became available in May 2023.^[4]^ In Japan, it is possible to undertake not only TKI but also ICI rechallenge, wherein patients are directly transferred to another ICI without any other systemic therapy, including TKI, as second-line treatment after the initial ICI.^[4]^ The American Association for the Study of Liver Diseases National Comprehensive Cancer Network clinical practice guidelines recommend TKI as second-line treatment,^[5,6]^ whereas there are few reports of the efficacy of ICI rechallenge.^[7,8]^ The efficacy of ICI rechallenge has been reported in solid tumors, such as malignant melanoma^[9]^ and renal cell carcinoma.^[10,11]^ However, the efficacy and safety of ICI contiguous rechallenge in uHCC needs evaluation. In the present study, we evaluated the efficacy and safety of Dur+Tre as the second-line regimen after first-line Atezo+Bev treatment in patients with uHCC.

## 2. Methods

### 2.1. Study design and participants

In this retrospective study, we enrolled patients with radiologically and/or histologically diagnosed HCC who received Atezo+Bev and Dur+Tre as the first- and second-line systemic therapy, respectively, at Hiroshima University Hospital, Hiroshima, Japan from November 2020 to July 2023. All participants had uHCC that could not be treated with surgery, catheterization, or ablation therapy; had not received TKI or other systemic therapy prior to Atezo+Bev; and had received at least one dose of Dur+Tre after the completion of Atezo+Bev treatment and without receiving any other systemic therapy. Clinical data, including baseline characteristics, treatments, response evaluations, and toxicity reports, were retrieved retrospectively from electronic medical records. All participants provided written informed consent to participate in the study, which was approved by the Hiroshima University Institutional Review Board, and this retrospective study was approved by the Ethics Committee of the Hiroshima University.

### 2.2. Treatment schedule

The patients received atezolizumab (1200 mg) with bevacizumab (15 mg/kg body weight) intravenously every 3 weeks. After cessation of Atezo+Bev therapy, the next therapy consisted of tremelimumab (300 mg) once and durvalumab (1500 mg) every 4 weeks. Patients who transiently or permanently discontinued either atezolizumab or bevacizumab because of an adverse event were allowed to continue monotherapy with either drug, as long as the attending physician determined that there was a clinical benefit. Furthermore, Atezo+Bev and Dur+Tre were continued even with disease progression if the attending physician determined that the patient derived treatment benefits. In cases with intrahepatic lesions refractory to systemic therapy, if the attending physician ascertained that the lesions could be controlled with on-demand transcatheter arterial chemoembolization (TACE), then TACE was performed without stopping systemic therapy. Dosing was continued until the attending physician determined that the patient could no longer benefit from treatment owing to disease progression or until an adverse event (AE) hindered treatment continuation.

### 2.3. Disease evaluation (efficacy)

The radiological response of HCC was assessed using contrast-enhanced computed tomography or magnetic resonance imaging and then evaluated by the investigators according to the Modified Response Evaluation Criteria in Solid Tumors.^[12]^ The best objective response (BOR) was defined as the best response according to the Modified Response Evaluation Criteria in Solid Tumors assessment compared with baseline. The ORR was defined as the percentage of patients with complete response (CR) or partial response (PR), and the disease control rate (DCR) was defined as CR, PR, or stable disease (SD). The first imaging evaluation was performed 4 weeks after treatment initiation, and imaging evaluations were scheduled every 8 weeks thereafter.

### 2.4. Treatment-related adverse events (safety)

Treatment-related adverse events (TRAE) and immune-related adverse events (IrAE) were graded using the National Cancer Institute Common Terminology Criteria for Adverse Events (V.5.0). Evaluations were performed at the time of ICI administration and upon the occurrence of an AE.

### 2.5. Statistical analysis

Intergroup differences in continuous and categorical variables were tested using the Mann–Whitney *U* or Fisher’s exact test, respectively. Paired *t* tests were conducted between each of the ALBI score. Statistical analysis was performed using JMP Pro17.0 (SAS Institute Inc., Cary, NC). *P* < .05 was considered statistically significant.

### 2.6. Data availability statement

Raw data were generated at Hiroshima University Hospital. Derived data supporting the findings of this study are available from the corresponding author A.O. on request.

## 3. Results

### 3.1. Clinical characteristics

**Figure 1.**
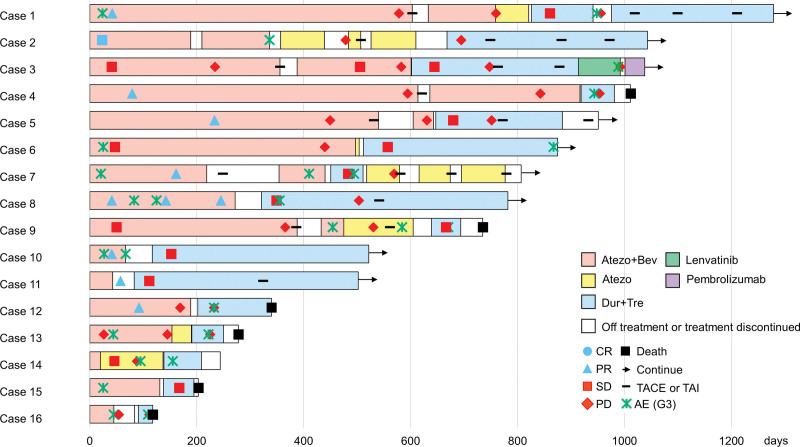
Swimmer plot describing the treatment course, date of onset of adverse events, radiological response, and response date of 16 cases. Colored solid bars: red, Atezo+Bev administration period; yellow, Atezo administration period; blue, Dur+Tre administration period; white, untreated period. Blue circle: CR. Blue triangle: PR. Red squares: SD, Red rhombi: PD. Black square: Death. Arrow: Ongoing treatment. Hyphen-Minus; TACE or TAI. Asterisk: Adverse events of CTCAE Grade 3. Atezo+Bev = atezolizumab–bevacizumab combination therapy, CR = complete response, CTCAE = Common Terminology Criteria for Adverse Events, DCR = disease control rate, Dur+Tre = durvalumab–tremelimumab combination therapy, ORR = overall response rate, PD = progressive disease, PR = partial response, SBRT = stereotactic body radio therapy, SD = stable disease, TACE = transarterial chemoembolization, TAI = transarterial infusion chemotherapy.

Sixteen participants were included in this study. All participants received Atezo+Bev as first-line therapy; 11 participants discontinued Atezo+Bev owing to progression of disease (PD), and 5 discontinued due to AEs. All participants chose Dur+Tre as the second-line systemic therapy. Participant demographics and clinical characteristics at the time of Atezo+Bev and Dur+Tre initiation are summarized in Table 1. The median age at the time of Atezo+Bev and Dur+Tre treatment initiation was 71 and 72 years, respectively; 12 and 6 patients were male and had a viral etiology, respectively. On the Child–Pugh liver function scale, 12 and 4 patients were classified as A and B at Atezo+Bev treatment initiation, whereas 8, 5, and 3 patients were classified as A, B, and C, respectively, at Dur+Tre treatment initiation, with worsening at Dur+Tre treatment initiation compared to Atezo+Bev. At Atezo+Bev initiation, the main tumor median diameter was 33.5 mm, and 9, 5, and 5 participants had multiple intrahepatic lesions, positive microvascular invasion, and extrahepatic metastasis, respectively. The Barcelona Clinic Liver Cancer stage was A, B, and C in 3, 4, and 9 participants, respectively. At Dur+Tre initiation, the median main-tumor diameter was 29.5 mm, and 9, 6, and 4 participants had multiple intrahepatic tumors, positive microvascular invasion, and extrahepatic metastases, respectively. The Barcelona Clinic Liver Cancer stage was A, B, and C in 3, 4, and 9 participants, respectively. The median α-fetoprotein level was 29.15 IU/mL at Atezo+Bev treatment initiation, but increased to 97.5 IU/mL at Dur+Tre initiation. The clinical courses of the 16 patients are shown in Figure 1. The median observation duration was 245.5 and 96.5 days for the Atezo+Bev and Dur+Tre groups. All participants received at least one dose of Atezo+Bev or Dur+Tre, and 11 of the 16 participants were advanced to Dur+Tre because of disease progression. Five patients discontinued Atezo+Bev due to AEs (proteinuria, 2 patients; fatigue, 2 patients; hand-foot syndrome, 1 patient). Owing to bevacizumab-related AEs (proteinuria: 4 patients, edema, hyperammonemia, and skin ulcer: 1 patient), 7 out of 16 participants switched to atezolizumab monotherapy. Similarly, 7 out of 16 participants underwent on-demand TACE while receiving Atezo+Bev and discontinued Dur+Tre. Six participants died, five of whom died of the primary disease.

**Table 1:** Participant characteristics stratified by treatment.

Variables	Atezo+Bev (n = 16)	Dur+Tre (n = 16)
Age (yr), median ± SD	71 (2.56)	72 (2.58)
Sex, male (%)	12 (75)	
Viral etiology (%)	6 (37.5)	
Child–Pugh stage (%)		
A	12 (75)	8 (50)
B	4 (25)	5 (31.3)
C	0 (0)	3 (18.8)
ALBI score, median ± SD	−2.68 (0.15)	−1.86 (0.17)
mALBI grade (%)		
1	9 (56.2)	5 (31.3)
2a	1 (6.3)	0 (0)
2b	5 (31.3)	7 (43.8)
3	1 (6.3)	4 (25)
Treatment prior to Atezo+Bev		
Surgery or ablation	5 (31.3)	
TACE or TAI	9 (56.3)	
Radiation	2 (12.5)	
Tumor diameter (mm), median ± SD	33.5 (10.2)	29.5 (38.3)
Multiple locations (%)	9 (56.3)	9 (56.3)
Macrovascular invasion (%)	5 (31.2)	6 (37.5)
Extrahepatic metastasis (%)	5 (31.2)	4 (25)
BCLC stage (%)		
A	3 (18.9)	3 (18.9)
B	4 (25)	4 (25)
C	9 (56.3)	9 (56.3)
Alpha-fetoprotein (IU/mL), median ± SD	29.15 (699.56)	97.5 (1919.3)
Reason for discontinuation of Atezo+Bev (%)		
Radiological progression	11 (68.8)	
Toxicity	5 (31.3)	

ALBI = albumin-bilirubin, Atezo+Bev = atezolizumab–bevacizumab combination therapy, BCLC = Barcelona-Clinic Liver Cancer, Dur+Tre = durvalumab–tremelimumab combination therapy, mALBI = modified albumin-bilirubin, TACE = transarterial chemoembolization, TAI = transarterial infusion chemotherapy.

### 3.2. Efficacy

**Figure 2.**
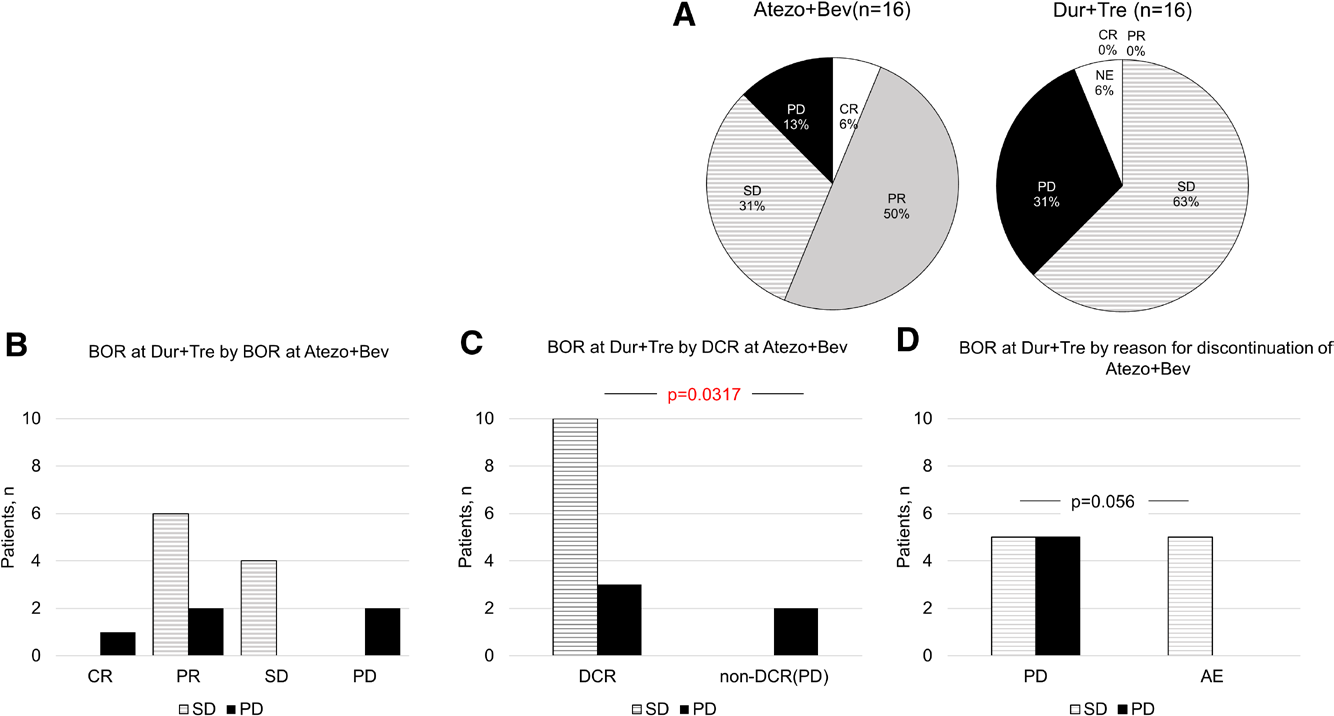
BOR at Atezo+Bev and Dur+Tre in 16 cases of the cohort. (A) BOR at Atezo+Bev and Dur+Tre, (B) BOR at Dur+Tre according to BOR at Atezo+Bev, (C) BOR at Dur+Tre according to DCR at Atezo+Bev, and (D) BOR at DUR+TRE by reason of discontinuation of Atezo+Bev. The significance threshold was set at a *P* ≤ .05. Atezo+Bev = atezolizumab–bevacizumab, CR = complete response, DCR = disease control rate, Dur+Tre = durvalumab–tremelimumab, NE = not evaluable, PD = progression of disease, PR = partial response, SD = stable disease.

**Figure 3.**
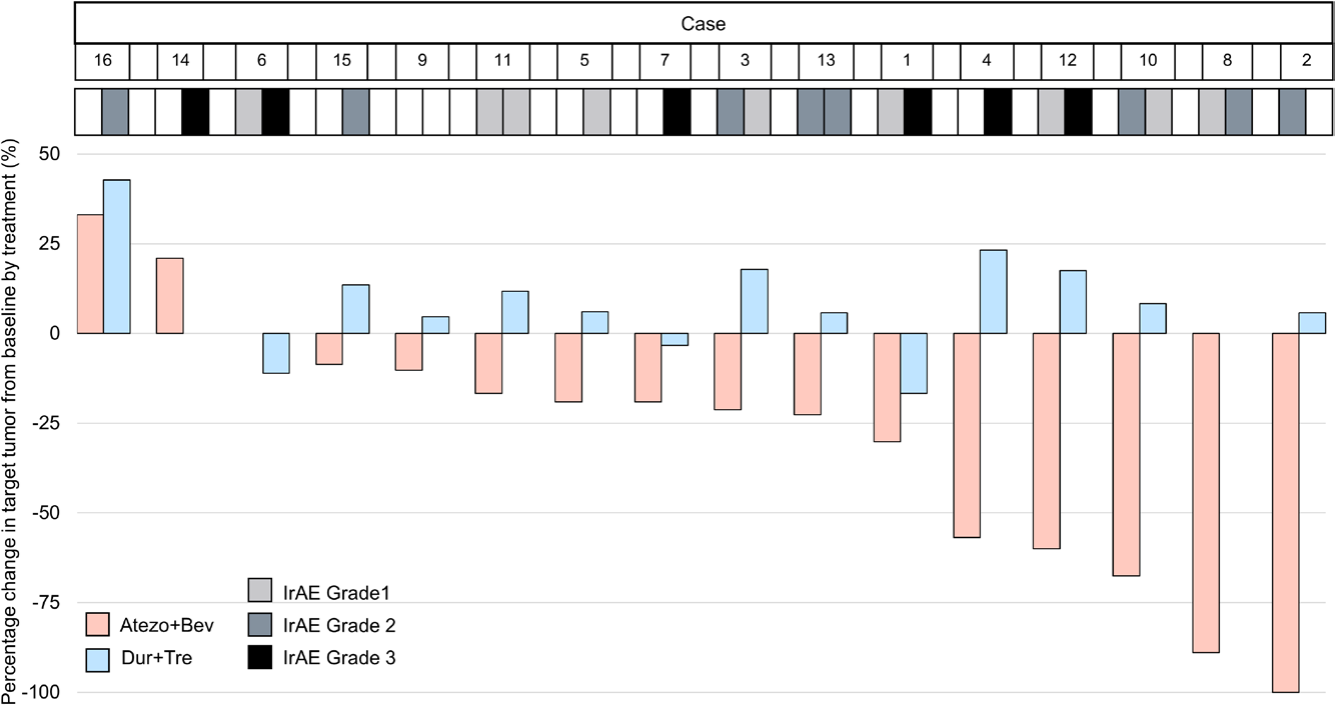
Waterfall plots of the percentage in target tumor from baseline by treatment. The *y*-axis represents the treatment-induced change in the target lesion from baseline. Treatments have been differentiated using different threads and irAEs are displayed on a color scale. Negative/positive values represent maximum tumor reduction or minimum tumor increase, respectively. Colored solid bars: red indicates Atezo+Bev and blue indicates Dur+Tre. Colored scale represents the CTCAE grade of irAEs. Atezo+Bev = atezolizumab–bevacizumab combination therapy, CR = complete response, CTCAE = Common Terminology Criteria for Adverse Events, DCR = disease control rate, Dur+Tre = durvalumab–tremelimumab combination therapy, IrAE = immune-related adverse event, PD = progression of disease, PR = partial response, SD = stable disease.

The efficacy, including BOR, ORR and DCR, of the Atezo+Bev and Dur+Tre treatments is shown in Table 2 and Figure 2 (Atezo+Bev, BOR: CR, 1; PR, 8; SD, 5; and PD, 2; ORR and DCR: 58.3% and 87.5%, respectively; Dur+Tre, BOR: SD, 10; PD, 5; there were no responders; DCR: 62.5%). In 1 case of Dur+Tre treatments, computed tomography could not be imaged, so the radiological response could not be assessed. Participants with PD during Atezo+Bev had PD during Dur+Tre. In the sub-analysis based on the reason for discontinuation, among participants who discontinued Atezo+Bev due to PD, during Dur+Tre, half of the patients had PD. In contrast, in all 5 cases with Dur+Tre discontinuation due to AE, the BOR was SD. The relationship between the maximum reduction rate of the target lesion and IrAEs for Atezo+Bev and Dur+Tre is shown in Figure 3. No correlation was observed between the Atezo+Bev and Dur+Tre groups.

**Table 2:** Clinical outcomes of participants in the treatment sub-cohorts.

Outcomes	Atezo+Bev (n = 16)	Dur+Tre (n = 15)
Best overall response (%)		
CR	1 (6.3)	0 (0)
PR	8 (50)	0 (0)
SD	5 (31.3)	10 (62.5)
PD	2 (12.5)	5 (31.3)
NE	－	1 (6.3)
ORR (CR+PR) (%)	9 (56.3)	0 (0)
DCR (CR+PR+SD) (%)	14 (87.5)	10 (62.5)

CR = complete response, DCR = disease control rate, NE = not evaluable, ORR = overall response rate, PD = progression of disease, PR = partial response, SD = stable disease.

### 3.3. Safety

**Figure 4.**
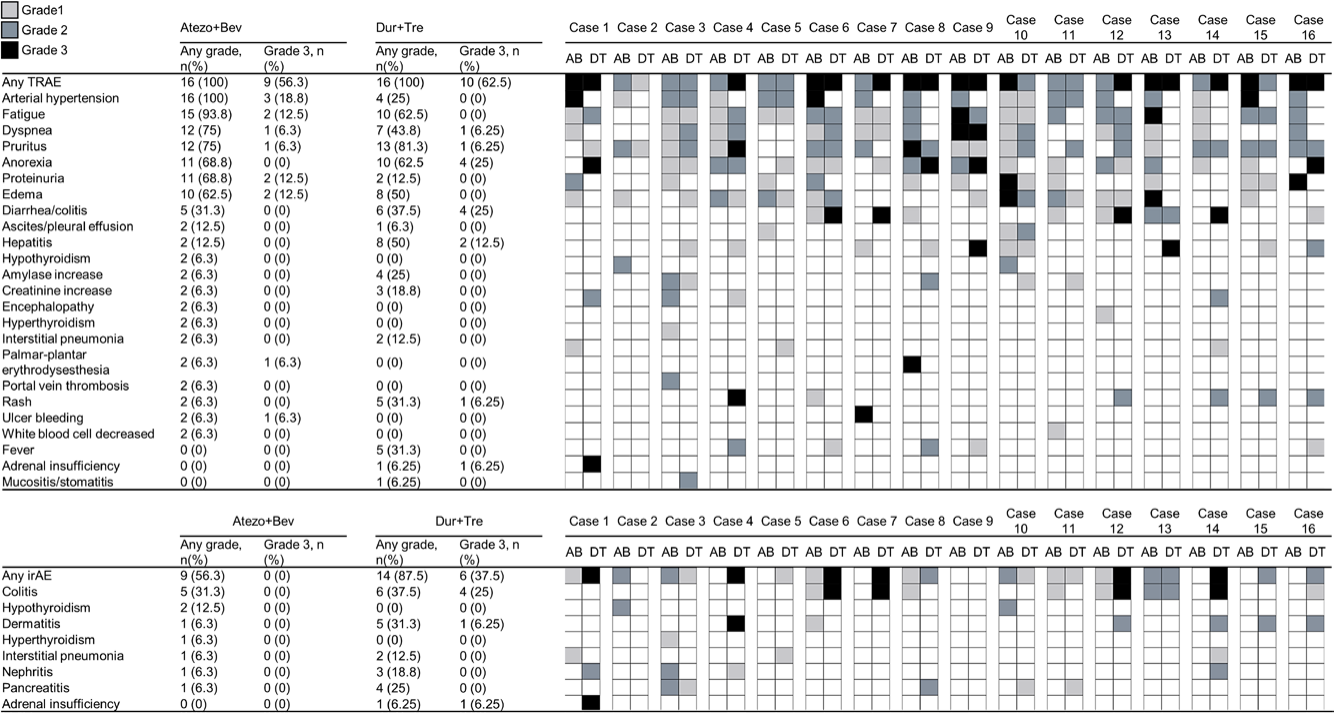
TRAE and irAE at Atezo+Bev and Dur+Tre in 16 cases of the study cohort. List of TRAEs and irAEs that occurred during the observation period. The CTCAE grade for each case is displayed on a color scale. AB = atezolizumab–bevacizumab combination therapy, CTCAE = Common Terminology Criteria for Adverse Events, DT = durvalumab–tremelimumab combination therapy, IrAE = immune-related adverse events, TRAE = treatment-related adverse events.

**Figure 5.**
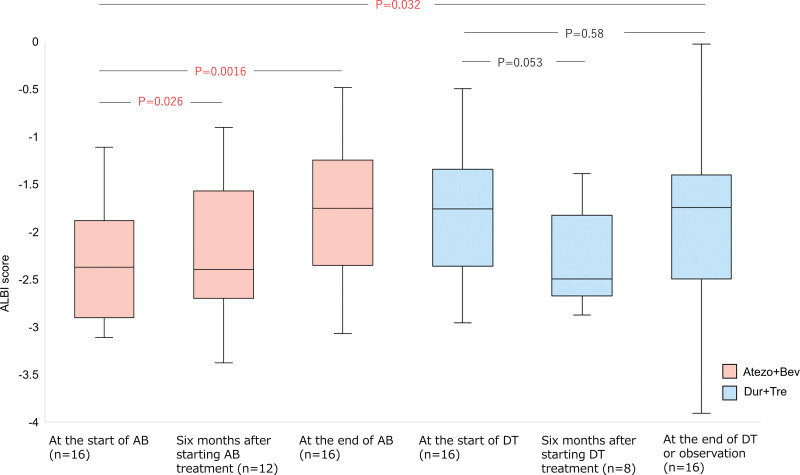
Box plot of changes in the ALBI score during the observation period. The BOX plot depicts the change in the ALBI score from the start of Atezo+Bev, 6 mo after the start of Atezo+Bev treatment, at the end of Atezo+Bev, at the start of Dur+Tre, at the end of Dur+Tre, or at the end of observation. The significance threshold was set at *P* ≤ .05. ALBI = albumin–bilirubin, Atezo+Bev = atezolizumab–bevacizumab combination therapy, Dur+Tre = durvalumab–tremelimumab combination therapy.

The TRAE and IrAEs associated with Atezo+Bev and Dur+Tre administration are shown in Figure 4. Regarding TRAEs, all patients with Atezo+Bev developed any AE, and 9 (56.2%) had Grade 3 AEs; the most common AE was hypertension (16 patients), followed by fatigue (15 patients) and dyspnea/pruritus (12 patients). Grade 3 AEs included hypertension (3 patients), fatigue, urinary protein, and edema (2 patients). Sixteen patients (100%) with Dur+Tre had any AE, and 10 (62.5%) experienced Grade 3 AE; the most common AE was itching (13 patients), followed by anorexia (10 patients) and fatigue (10 patients). The most common Grade 3 AE was anorexia and diarrhea (4 patients). Nine (56.3%) of the Atezo+Bev patients developed an IrAE and the most common irAE was colitis (5 cases); no Grade 3 or higher IrAEs were observed during Atezo+Bev administration. In the Dur+Tre group, 14 patients (87.5%) developed any IrAE, and Grade 3 IrAEs occurred in 6 patients (37.5%). The maximum grade of IrAEs during Dur+Tre administration was Grade 3, and no Grade 4 or higher IrAEs were observed. Of the 3 patients who had colitis with Atezo+Bev, 3 (60%) also had colitis with Dur+Tre, and 2 (40%) had exacerbations. Regarding other IrAEs, only 1 case of elevated pancreatic enzymes was observed as a common IrAE in Atezo+Bev and Dur+Tre; in many cases, no involvement was found in IrAE onset with Atezo+Bev and Dur+Tre. There were no serious irAEs requiring steroid treatment or withdrawal during Atezo+Bev treatment, but 1 case each of colitis and adrenal insufficiency requiring steroids and withdrawal during Dur+Tre treatment. The changes in ALBI scores are shown in Figure 5. After starting Atezo+Bev, there was a significant worsening of the ALBI score at 6 months and at the time of discontinuation; however, no significant worsening was observed at the start of Dur+Tre treatment and at 6 months and at the end of treatment discontinuation and observation.

## 4. Discussion

Owing to a lack of data on the use of subsequent ICIs in patients who have been previously treated with an ICI,^[5]^ the National Comprehensive Cancer Network guidelines indicate ICIs only for patients who have not been previously treated with checkpoint inhibitors. This is because the extant efficacy evaluations of ICIs as second-line therapy have been undertaken post-sorafenib, and no prospective studies on ICI rechallenge have been published.^[2,3,13–15]^ According to the American Association for the Study of Liver Diseases guidelines, Atezo+Bev is recommended for the treatment of advanced- or intermediate-stage HCC, excluding liver transplant and autoimmune disease cases, whereas Dur+Tre is indicated only for cases with a high risk of gastrointestinal bleeding.^[6]^ TKIs are recommended as second-line treatments, and ICIs, such as nivolumab–ipilimumab or pembrolizumab, can only be used in cases where TKI use is infeasible.^[6]^ Despite the limited indications for second-line ICI, 24.3% of patients who underwent second-line therapy after Atezo+Bev received ICI in clinical practice,^[16]^ and there is a great need to accumulate data on the safety and efficacy of ICI therapy. The currently available retrospective reports^[7,8]^ on ICI rechallenge for uHCC are limited to Atezo+Bev, nivolumab+ipilimumab, pembrolizumab, or clinical trials. Moreover, the participants of these previous studies included patients who received TKI between the first- and second-line immunotherapy. This is the first report of a rechallenge from Atezo+Bev to Dur+Tre that does not include systemic therapy other than ICIs, such as a TKI.

In our analysis of uHCC patients treated with Dur+Tre after Atezo+Bev treatment, the ORR for Atezo+Bev as first-line therapy was 56.3%, and the DCR was 87.5%; the ORR for Dur+Tre as second-line therapy was 0%, and DCR was 62.5%. Grade 3 AEs were present in 56.3% of Atezo+Bev patients and 62.5% of Dur+Tre patients. Although Grade 3 IrAEs were not observed with Atezo+Bev, these were seen in 37.5% of participants during the Dur+Tre treatment period. The most common IrAE in both Atezo+Bev and Dur+Tre was colitis; of the 5 patients with colitis in Atezo+Bev, 3 (60%) had colitis with Dur+Tre, and 2 (40%) had exacerbations.

The efficacy of ICI rechallenge in HCC has not been established despite various reports on the efficacy and safety of ICI rechallenge in cancers of other organs.^[10,17,18]^ For BOR of second-line ICI, the ORR and DCR were 10.8–25.0%^[9–11,19,20]^ and 19.2–64.0%, respectively.^[9–11,20]^ In all reports, the response rate for second-line ICI was lower than that for first-line ICI. The response rates for second-line IrAEs were 45–64% for all grades and 13–21%^[10,11,19]^ for Grade 3 IrAEs. There was no difference in the incidence of any grade IrAEs between ICI-1 and ICI-2 in either study, and there was no significant correlation between IrAEs that occurred with first- and second-line ICI.^[19]^ Colitis is one of the most common IrAEs, and 34% of participants who had colitis with first-line ICI had recurrent colitis with second-line ICI, of which 12% had Grade 3 or higher colitis.^[17]^ In fact, in the current study, although most AEs were not common between Atezo+Bev and Dur+Tre, patients relapsed with a high probability. A high recurrence rate of hepatitis and pneumonia has also been reported,^[21]^ suggesting that ICI rechallenge for patients with these IrAEs in the first-line ICI should be performed with caution.

The efficacy of ICI rechallenge in HCC has been reported, with a BOR of ORR 16–26% and DCR 52–55% for second-line ICI.^[7,8]^ However, 38% to 40% of patients received systemic therapy, including TKI before first-line ICI, and in addition, 29% to 40% of patients received systematic therapy other than ICI before second-line ICI.^[7,8]^ Therefore, these results do not indicate the effectiveness of a sequential ICI rechallenge. Some types of IrAEs occurred in 48% to 52% of cases with second-line ICI,^[7,8]^ among which the common IrAE were dermatitis, colitis, and liver damage, and 12% to 17% of patients experience Grade 3 or higher irAE.^[7,8]^ Only 3 patients experienced grade 3 or higher AEs with either the first-line ICI or the second-line ICI,^[7]^ and the tolerability was considered good. In our study, no uncontrollable irAEs were observed, and although no response was obtained, DCR was achieved in most patients. Based on this, although it is unlikely that success would be achieved with ICI rechallenge with Dur+Tre after Atezo+Bev, there was a possibility that the disease could be controlled relatively safely.

A unique feature of our study was the assessment of changes in ALBI score during the treatment period. Poor liver function not only limits treatment options for uHCC,^[22]^ but is also a poor prognostic factor: the ALBI score has been reported to be a poor prognostic factor in intermediate stage HCC and may reduce the response and prognosis of ICIs and TKIs.^[23]^ In our study, 6 patients remained on Dur+Tre for more than 180 days while maintaining liver function, despite the addition of other therapies such as TACE.

The efficacy of TKI after Atezo+Bev for uHCC was reported with ORR 6.1–33.0% and DCR 70.4–95.0%,^[24–27]^ which is a higher response rate than observed in this study. AEs included fatigue, anorexia, hand-foot syndrome, and proteinuria; and AEs of any grade were observed in 85.7% and grade 3 or higher in 16.3%.^[24–26]^ In particular, proteinuria was observed in several patients (16.8–40%).^[24–26]^ Patients with AE-related fatigue and loss of appetite tend to have a decline in liver function in the early stages of administration, and caution is required.^[25]^ According to our report on the efficacy of Len after Atezo+Bev, the ORR and DCR were 33.3% and 75.0%, respectively; regarding the response, 45.8% of patients had anorexia, 29.2% had proteinuria, and 71.4% of patients with proteinuria had grade 3 or higher proteinuria. Liver hypofunction can be a major obstacle for continued Len administration.^[27]^ In contrast, in this study, there was no significant decline in liver function during the period of Dur+Tre treatment, which may be a strength of Dur+Tre compared to second-line TKIs, as reserve capacity is less likely to decline with Dur+Tre.

Although disease control was achieved in many patients in the present study, no response was observed. To enhance the therapeutic effects of ICI rechallenge, it is necessary to reignite the immune microenvironment. Recently, combination therapies with locoregional therapies, including ablation, transarterial embolization, and radiotherapy, which are usually used for early-stage HCC, have been actively explored to enhance ICI efficacy by promoting the release of tumor-associated antigens and cytokines, eventually accelerating the so-called cancer-immunity cycle.^[28]^ In fact, the combination of ICI with TACE, ablation therapy, and TKI has high therapeutic efficacy,^[29–31]^ and the combination of TACE and ablation therapy to modulate the immune microenvironment after disease progression with ICI may enhance the therapeutic efficacy of ICI rechallenge. In a report on successful rechallenge from Atezo+Bev to Dur+Tre, the combination of Atezo+Bev with TACE was cited as a contributory factor for success.^[32]^ In this study, SD was achieved in 4 of the 7 cases in which Atezo+Bev was combined with TACE, although Atezo+Bev was discontinued in all 7 cases due to disease progression. In addition, we have reported that lenvatinib exerts immunomodulatory effects as well.^[33]^ Transcriptome analysis revealed that immune signatures associated with T-cell functions and interferon responses were enriched in the early phase of treatment, whereas signatures associated with immunoinhibitory cells were downregulated along with efficient vascular endothelial growth factor receptor and fibroblast growth factor receptor blockade after the 4-week lenvatinib treatment. Moreover, we performed imaging mass cytometry, which revealed that intratumoral TAM and Tregs significantly decreased after the 4-week lenvatinib treatment.^[33]^ Therefore, we consider LEN a possible option for systemic therapy in the interval between first- and second-line ICI. It is necessary to devise a treatment that induces changes in the microenvironment to achieve a response to ICI rechallenge. The goal of systemic therapy is not only response but also prolongation of survival, for which the maintenance of liver function is an important factor. To determine whether ICI rechallenge with or without intervening treatment, such as Len, it is necessary to analyze which, including post-treatment, provides better long-term OS.

One of the limitations of our study was the short duration of Dur+Tre treatment, and ORR might improve over longer treatment periods. In the HIMALAYA study, the time to response was 2.17 (95% CI 1.84–3.98) months,^[34]^ and the median observation period after the start of Dur+Tre in this study was 4.0 months, so it is possible that early cases were included in the efficacy assessment. Furthermore, IrAEs may occur in the future as the observation period is extended. In the HIMALAYA study, 74% of IrAEs occurred within 3 months of treatment.^[35]^ On the other hand, 12% of IrAEs occurred after 6 months of treatment, and 3.8% of cases were Grade 3 or higher IrAEs.^[35]^ In this study, IrAE occurred in 1 case after more than 200 days, and it is possible that more IrAEs will be observed as the observation period is extended. Another limitation was the small sample size and the retrospective nature of the study. Due to the small sample size, responder cases may not have been included in this study, but there have been reports of responses to salvage ipilimumab plus nivolumab.^[36]^ In addition, although other treatments were added, 9 patients survived and 6 were able to continue treatment, suggesting that it is possible that long-term OS will be achieved in the future. The DCR of Dur+Tre therapy was comparable to that of Sorafenib (60.1% and 60.7%, respectively); however, the 4-year survival rates were 25.2% and 15.1%, respectively, showing a significant OS benefit with Dur+Tre compared to Sorafenib (OS HR: 0.78 [96.02% confidence interval (CI) 0.65–0.93; *P* = .0035]).^[37]^ Moreover, the 4-year survival rates of Dur+Tre among patients with well-controlled disease were 36.2%, indicating a further improvement in OS.^[37]^ Direct sequential treatment with Dur+Tre after first-line Atezo+Bev treatment might be a potential treatment in terms of prolonging survival through improved liver reserve, but a large study would be needed to conclude this.

In conclusion, this study demonstrates that ICI rechallenge with Dur+Tre after Atezo+Bev is unlikely to be successful but may control the disease relatively safely.

## Acknowledgments

The authors thank all the medical workers who were involved in this study.

## Author contributions

**Conceptualization:** Atsushi Ono, Tomokazu Kawaoka.

**Data curation:** Shigeki Yano, Kei Amioka, Kensuke Naruto, Kenji Yamaoka, Yasutoshi Fujii.

**Investigation:** Shigeki Yano, Shinsuke Uchikawa, Tomokazu Kawaoka.

**Project administration:** Tomokazu Kawaoka.

**Supervision:** Atsushi Ono, Shigeki Yano, Hatsue Fujino, Takashi Nakahara, Eisuke Murakami, Tomokazu Kawaoka, Daiki Miki, Masataka Tsuge, Shiro Oka.

**Writing – original draft:** Ryoichi Miura.

**Writing – review & editing:** Atsushi Ono, C Nelson Hayes.
